# Assessment of intralesional cytokine profile of cutaneous leishmaniasis caused by *Leishmania donovani* in Sri Lanka

**DOI:** 10.1186/s12866-018-1384-4

**Published:** 2019-01-14

**Authors:** Lahiru Sandaruwan Galgamuwa, Buthsiri Sumanasena, Devika Iddawela, Susiji Wickramasinghe, Lalani Yatawara

**Affiliations:** 1 Department of Parasitology, Faculty of Medicine, University of Peradeniya, Peradeniya, 20400 Sri Lanka; 2 Anuradhapura Teaching Hospital, Harischandra Mawatha, Anuradhapura, Sri Lanka; 3 Department of Parasitology, Faculty of Medicine, University of Peradeniya, Peradeniya, 20400 Sri Lanka; 4 Department of Medical Laboratory Sciences, Faculty of Allied Health Sciences, University of Peradeniya, Peradeniya, 20400 Sri Lanka

**Keywords:** Cutaneous leishmaniasis, IFN-γ, IL-4, IL-11, IL-12p40, Sri Lanka

## Abstract

**Background:**

Cytokines play a vital role in the host immune response to infection by initiating the healing process and/or accelerating the progression of the disease in cutaneous leishmaniasis (CL). Very little evidence is available on cytokine profiles and their regulatory function in CL patients in Sri Lanka. The aim of this study was to determine the cytokine expression pattern of IFN-γ, IL-4, IL-11 and IL-12p40 in CL patients and in healthy volunteers. Patients with suspected CL lesions attending to the Dermatology Clinic at the Anuradhapura Teaching Hospital were included in the study. Reverse transcription real time polymerase chain reaction (real-time RT-PCR) was performed to determine the relative expression level of target cytokines. Expression levels were quantified by 2^- ∆∆CT^ equation.

**Results:**

The expression of cytokines IFN-γ, IL-4, IL-11 and IL-12p40 were significantly higher in CL patients compared to healthy volunteers (*p* <  0.05). There was a significant association between the expression of IFN-γ and the duration of the lesion (*p =* 0.021). Wet CL lesions showed significantly higher expression of IL-4, IL-11 and IL-12p40 (*p* = 0.039, 0.018 and 0.021 respectively) compared to dry lesions. Papulo-nodular lesions showed significantly high expression of IFN-γ (*p* = 0.023). However, cytokine expression was not significantly associated with the number, size and the locations of lesions.

**Conclusions:**

The expression levels of all cytokines tested in the present study were significantly (*p* <  0.05) high in CL patients. Th1 response (IFN-γ and IL-12p40) had higher expression levels compared to Th2 (IL-4) and IL-11 in CL patients.

## Background

Cutaneous Leishmaniasis (CL) is caused by an intracellular protozoan from the genus *Leishmania* that infects mononuclear phagocytes of mammals. According to the published data, an estimated 0.7–1.2 million new cases are documented globally, particularly in tropical and subtropical countries [1]. Leishmaniasis has diverse clinical manifestations (visceral, cutaneous and mucocutaneous) due to differences in interactions between the infecting species and the host’s immune response [2]. However, cutaneous leishmaniasis is the predominant clinical form found in Sri Lanka.

Cytokines play a vital role in the host immune response to *Leishmania* infection by directing the development of protective and non-protective immunities during the infection [3]. These inflammatory responses mediate disease progression and clinical outcome. Th1 type cellular immune responses induce the defense mechanism against *Leishmania* parasites, while activation of Th2 type cells results in progressive disease [4, 5]. According to McDowell et al. (2002), differences in the mechanism of interaction with phagocytic cells between different *Leishmania* species may result in healing or non-healing forms of the disease, dependent up on their ability to activate Th1 response [6]. The development of cell-mediated immune responses capable of controlling *Leishmania* infection and resolving disease result from signaling by interferon gamma (IFN-γ), secreted primarily by activated T cells (Th1) and natural killer (NK) cells in response to interleukin-12 (IL-12) signaling [7]. Resistance to infection by *Leishmania* parasites is mediated by interferon gamma (IFN-γ) that stimulates macrophages to produce nitric oxide (NO) which is essential for leishmanicidal activity [8]. IFN-γ also inhibits the production of cytokines such as IL-4, and IL-10 associated with Th 2 response. Increased expression of IL-4 and IL-10 was found to be linked with failed healing and disease progression [5, 9].

IL-4 plays a major role in the non-healing response observed following *Leishmania* infection by down-regulating the expression of protective Th1 associated cytokines (IL-12 and IFN-γ) and by inhibiting NO production [10]. Several studies have shown that Th1 response was prominent in the healing form while Th2 was the prominent response in non-healing forms of CL [11, 12]. These observations suggest that the balance between Th1 and Th2 cytokine profiles may decide the development of visceral or cutaneous disease: a prominent Th1 response leads to the cutaneous form, while a predominant Th2 response leads to visceral disease. IL-11 is a member of the IL-6 family and is produced by bone marrow stromal cells [13]. It promotes differentiation of progenitor B cells and megakaryocytes. It decreases Th1 cell differentiation and inhibits the production of proinflammatory cytokines including TNF-α, IL-1β and IL-12p40 [14] while enhancing Th2 responses [15].

In Sri Lanka, CL is caused by *Leishmania donovani* zymodeme MON-37 [16]. DNA sequencing and microsatellite analyses have shown that Sri Lankan isolates are closely related to those causing visceral leishmaniasis (VL) in the Indian subcontinent [17]. *L. donovani* is known to cause a visceral disease mainly in other countries, though cutaneous form has also been reported [16, 18]. Many immunological studies have been carried out on CL caused by *L. major*. However, very few studies have been conducted on the immunological response of CL caused by *L. donovani* in Sri Lanka. Therefore, analysis and quantification of cytokine response in Sri Lankan patients are of vital importance in explaining how immune responses contribute to the disease progression. In the present study, messenger ribonucleic acid (mRNA) expressions of IFN-γ, IL-4, IL-11 and IL-12p40 were evaluated in CL patients and compared with healthy volunteers.

## Methods

### Patients and samples

This study was carried out from January to July 2015. Patients attending to the Dermatology Clinic in Anuradhapura Teaching Hospital with suspected CL lesions were enrolled in this study. The purpose of the study was explained to the participants who consented to enroll in the study. Skin biopsy samples were obtained from 43 CL patients without the history of any previous treatment for leishmaniasis. Ten healthy volunteers without previous history of CL were enrolled as controls. Skin biopsies and slit skin smears were analyzed on each patient for cytokine assay and identification of *Leishmania* amastigotes. Pregnant women and patients with a history of chronic diseases or previous treatment with anti leishmaniasis drugs were excluded from the study.

### Control group

Ten healthy volunteers (mean age 37.3 ± 14.0 years, male: female – 5:5) with no history of leishmaniasis were recruited as controls and skin biopsies were obtained to assess the cytokine profiles.

### Data collection

Patients’ demographic data and the characteristics of lesions were recorded using a patient information sheet. Recorded characteristics of lesions include duration (from onset of lesion to the time of presentation in months), site, size, and number of lesions (single or multiple). Types of lesions were classified as papulo-nodular, nodulo-ulcerative, ulcerative lesions. Elevated lesion with no visible fluid with a diameter of 0.5 cm or more was categorized as a papulo-nodular lesion. Small glistering, translucent skin over a colored papulet or acentral ulcer with raised glistening edges was categorized as a nodulo-ulcerative lesion and discontinuation of the epithelial lining extending into the epidermis/dermis was categorized as an ulcerative lesion [19].

Lesions were further categorized into wet (characterized by sero-purulant exudates, redness, and inflamed open margins) and dry (covered by a crust or a scab) types of lesions.

### Diagnosis of cutaneous leishmaniasis

The diagnosis of CL was performed by direct microscopic observation of amastigotes in stained smears and/or by polymerase chain reaction (PCR). CL infection was confirmed by the positive findings from one of the either methods (microscopy or PCR).

### Collection of slit skin smears and detection of *Leishmania* amastigotes

The lesion along with surrounding skin was cleaned with 70% ethanol and allowed to dry. A small incision was made in the active edge of the lesions with the point of a sterile lancet. The lancet was then turned 90° and scraped along the cut edge of the incision to pick up the tissues and tissue fluid which was then smeared on cleaned glass microscope slide. Three to five smears were obtained from discrete areas of the lesion. When a patient had multiple lesions, samples were obtained from each lesion separately. Smears were then air dried and fixed with 100% methanol, stained with Giemsa stain after which they were examined under light microscope using (X100) magnification to detect *Leishmania* amastigotes.

### Collection of biopsy specimens

Adrenaline with lignocaine (0.1 ml) was used for local anesthesia at the site of biopsy. A circular piece of skin with a dermal thickness of 3 mm was taken from the border of the ulcer using Visipunch™biopsy needle. The biopsy was immediately frozen in liquid nitrogen and an identification number was assigned for each sample. These biopsy samples were transported to the Department of Parasitology, Faculty of Medicine, University of Peradeniya on the same day and stored in liquid nitrogen until use.

### Polymerase chain reaction

All biopsy samples were subjected to PCR to identify causative *Leishmania* species. Deoxyribo nucleic acid (DNA) was extracted from each biopsy sample according the manufacturer’s protocol of Pure Link ™ Genomic DNA Mini Kit (Invitrogen, Life Technologies, USA). Extracted DNA was analyzed by PCR using primers specific for all old world *Leishmania* spp. [20]. Then, positive samples were subjected to nested PCR using *Leishmania donovani* specific primers to amplify kinetoplast DNA (kDNA) [21]. Precautions were taken during the preparation of PCR reaction mixture to prevent any contamination. PCR products were visualized on 1.5% agarose gels. Positive and negative controls were included in each PCR reaction. DNA extracts of CL positive samples stored in − 80 °C freezer were used as positive controls. Distilled water was used as negative controls.

### RNA extraction

RNA was extracted using SV total RNA isolation kit (Promega, USA) according to the manufactures instruction. The integrity of all purified RNA was evaluated by 1% agarose gel electrophoresis by maintaining the intensity of 28S to18S rRNA at approximately 2:1 ratio. Total extracted RNA from each sample was measured by the QuantiFluor-ST RNA system (Promega). Complementary DNA (cDNA) was synthesized using a SuperScript™ II Reverse Transcriptase kit (Invitrogen) using 1 microgram (μg) of total RNA.

### Real-time PCR assay and quantification of cytokine mRNA expression

Equal amount of 100 nanograms (ng) of cDNA templates was used in each PCR reaction for quantification of cytokine mRNA. PCR master mixture was prepared using GoTaq®qPCR Master Mix (Promega). The cDNA templates were then added to 40 μl (μl) reaction mixture containing 25 μl of GoTaq®qPCR Master Mix (Promega) along with forward and reverse primers at a final concentration of 200 nM and 10 μl RNase free H_2_O. Published oligonucleotide sequences of glyceraldehyde-3-phosphate dehydrogenase (GAPDH) gene (control) and cytokine genes of IFN-γ, IL-4, IL-11 and IL-12p40 were analyzed in the study [22, 12]. Details of primers and probes used for amplification of cytokines were shown in Table 1. Real-time quantitative PCR was performed by a Rotor Gene Q system (Qiagen, Germany). The cycle was programmed for initial activation of Hot-Start *Taq* polymerase at cycling parameters of 95 °C for 2 min, 40 cycles of denaturation at 95 °C for 15 s and combined annealing and extension at 60 °C for 1 min.

**Table 1:** Oligonucleotide sequence of primers used for amplification of cytokines

Cytokine	Oligonucleotides sequence (5′—› 3′)	Fragment size (bp)
IFN –γ	F: CAG CTC TGC ATC GTT TTG GGT TCT	466
	R: TGC TCT TCG ACC TCG AAA CAG CAT	
IL-11	F: CACATGAACTGTGTTTGCCGCCTGGT	295
	R: GCAGCCTTGTCAGCACACCTGGGAGCTGTAGA	
IL – 4	F: GAA CAG CCT CAC AGA GCA GAA GAC	221
	R: TGT CGA GCC GTT TCA GGA ATC	
IL-12p40	F: CCA AGA ACT TGC AGC TGA AG	355
	R: TGG GTC TAT TCC GTT GTG TC	
GAPDH	F: TGACCTCAACTACATGGTTTA	151
	R: GCCCCACTTGATTTTGGA	

For RT-PCR, standard dissociation curves were constructed for housekeeping and cytokine genes. A single dissociation peak was obtained for the product of each primer set. It was confirmed that RT-PCR assay was gene specific and the results obtained were not confounded by nonspecific amplification or primer dimers.

The slopes of standard calibration plots of the mean cycle threshold (C_T_) values versus log genomic DNA concentrations for serial dilutions of 200 to 3.125 μM were − 2.97 for IFN-γ, − 3.22 for IL-4, − 3.02 for IL-11, − 3.45 for IL-12 p40 and − 3.15 for GAPDH respectively. The mean correlation (r^2^) of detection of all transcripts was 0.937 for IFN-γ, 0.978 for IL-4, 0.972 for IL-11, 0.954 for IL-12 p40 and 0.981 for GAPDH respectively.

Comparative C_T_ method was used to calculate differences in gene expression values. C_T_ value was defined as the number of PCR cycles required for the florescence signal to exceed the detection threshold value. cDNA was normalized with GAPDH products to compare the expression of each cytokines. Three master mixtures were made from each sample and average C_T_ values were taken. Expressions of mRNA of each gene were quantified by means of 2 ^-∆∆CT^ equation and the relative expressions of cytokine genes of IFN-γ, IL-4, IL-11 and IL-12p40 were calculated for each CL patients and healthy individuals.

### Statistical analysis

Data from the questionnaire and C_T_ values obtained from real-time PCR instrumentation was entered into a Microsoft Excel sheet and transferred to SPSS version 17 statistical software (SPSS Inc., Chicago, IL, USA) for statistical analysis. Nonparametric tests were used in this study since the expressions of mRNA values were not normally distributed with clinical evaluations. The expressions of cytokine mRNAs were considered as dependent variables while clinical variables were considered as independent variables in these tests. Individual immunological data in more than two groups was compared with each other by applying the Kruskal–Wallis test. This ranked the mean values of two or more studied groups and then assessed the variations of these ranks to determine the significant difference between groups. Correlations between the expressions of cytokine mRNAs were calculated by the Spearman’s correlation test. Pair-wise comparison of target gene expression was analyzed by the Mann-Whitney U-test and the results are expressed as medians and interquartile ranges.

The expressions of cytokines were transformed from continuous to categorical using logarithms. Multiple regression was applied to predict a model between the expression of each cytokines, association of various socio-demographic and clinical variables with log transformed data. Relationship between the informed data of patients (age, sex, ethnic group, clinical presentation, details of the lesion) and their production of cytokines were presented as mean (±) standard error (SE) and a difference in mean values were considered significant when the *p-*value is < 0·05.

### Ethical considerations

The procedure was approved by the Ethics Review Committee, Faculty of Medicine, University of Peradeniya, Sri Lanka. Permission to conduct the study was obtained from the administration of the Anuradhapura Teaching Hospital. All the participants were informed that their participation was voluntary and they had the right to withdraw from the study at any given time. Written informed consent describing the purposes and procedures was obtained from each adult participant and from the parents or the legal guardian of children less than 18 years old. The information provided to patients was written in their native language. All information provided by the respondents was kept confidential and used only for the purpose of the study.

## Results

### Clinical profile of the study participants

**Fig. 1:**
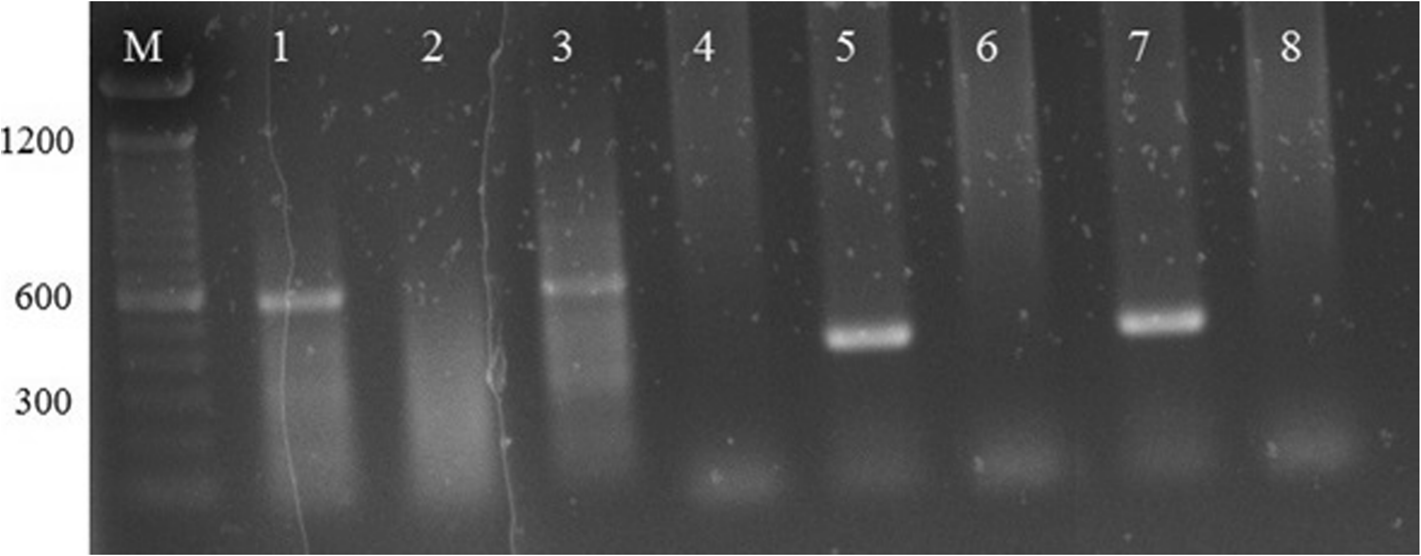
Gelelctrophoresis of nested PCR products of species specific primers of Leishmania donovani. M – molecular marker (100 bp), Lanes 1 to 4 – First PCR products, Lanes 5 to 8 – Second PCR products, Lanes 1 and 5 – Positive control, Lanes 2,4,6 & 8 – Negative controls, Lanes 3 and 7 – Test sample. Expected fragment size for the 1st PCR was 576 bp and 2nd PCR was 378 bp

Forty three patients with suspected leishamaniasis (mean 37.8 ± 12.6 years, male: female - 33:10) and 10 healthy volunteers (mean 37.3 ± 14.0 years, male: female – 5:5) were recruited into this study. Amastigotes were observed in only 38 Giemsa stained samples. However, positive results were obtained from all 43 samples by nested PCR. Nested PCR results confirmed the *Leishmania donovani* as the etiological agent responsible for CL in this study group. Figure 1 shows results of nested PCR. Number of lesions per patient varied from 1 to 3 (mean 1.3 ± 0.5) and the surface area of lesions ranged from 22 to 1800 mm^2^ (mean 256.1 ± 102.4). The duration of lesions were varied from 2 weeks to 54 months. Most lesions were found in exposed areas of the body with the highest number of lesions in upper and lower limbs. Nodular-ulcerative and dry type of lesions were the commonest type of lesions observed among CL patients (Table 2).

**Table 2:** Basic clinical characteristics of leishmaniasis patients

Variables	Frequency
Sex (male/female)	33/10
Age, mean (SD) years	37.8 (12.6)
Duration of lesions, mean (SD) months	10.5 (6.8)
No. of lesions, mean (range)	1.3 (1–3)
Size of lesions, mean (range) mm^2^	256.1 (22–1800)
Site of lesions	
Upper limbs	20 (46.5%)
Lower limbs	14 (32.6%)
Face/Neck	9 (20.9%)
Type of lesions	
Papulo-nodular	15 (34.9%)
Nodular-ulcerative	20 (46.5%)
Ulcers	8 (18.6%)
Dry lesions	32 (74.4%)
Wet lesions	11 (25.6%)

### Intralesional expression of cytokine mRNAs

**Fig. 2:**
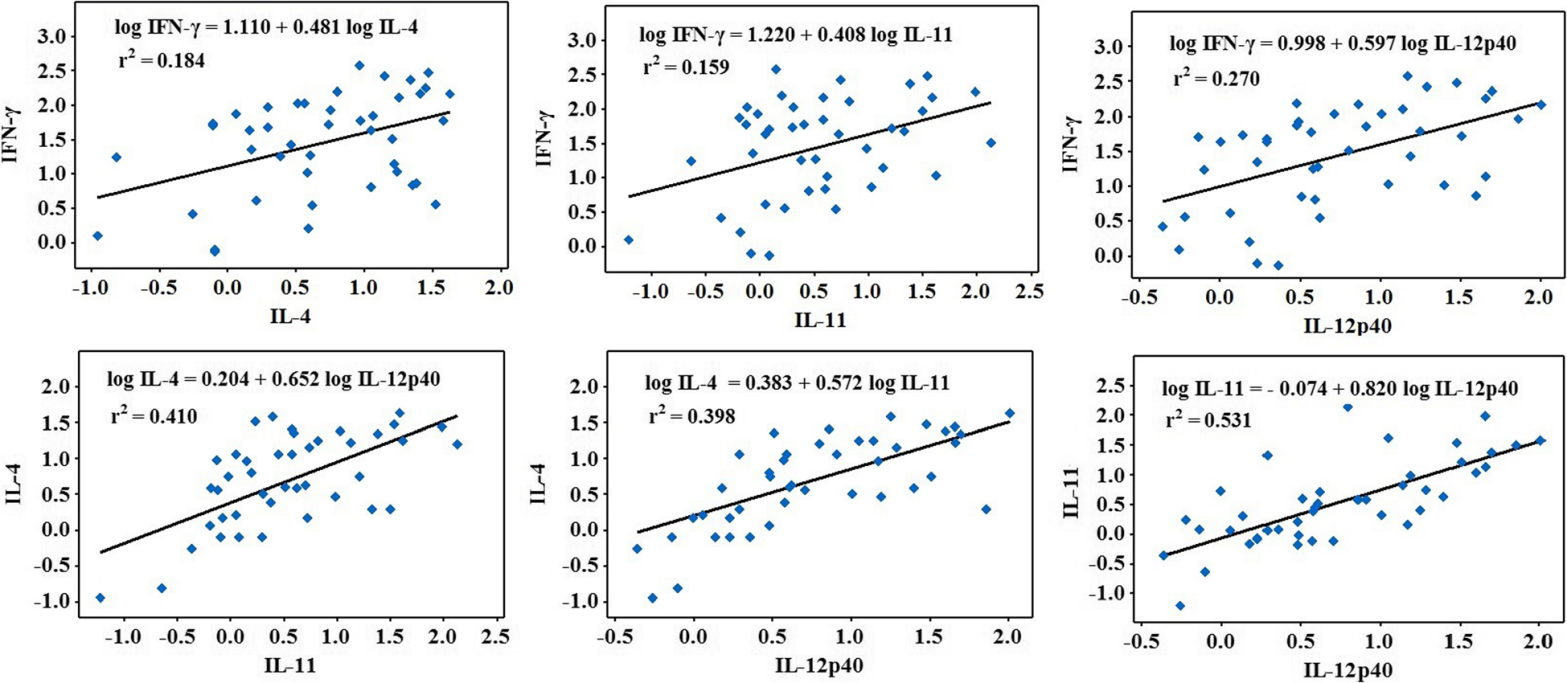
Correlation graphs of log normalized cytokine expressions in CL patients. x and y values - log_10_ normalized gene expressions

mRNA expression of IFN-γ, IL-4, IL-11, IL-12p40 cytokines and GAPDH was analyzed by RT-PCR. Expression levels of cytokine mRNA was varied from lesion to lesion within a wide range (Fig. 2). The expressions of Th1-associated cytokines (IL-12 and IFN-γ) were significantly higher than Th2-associated cytokines (IL-4). The highest expression was observed in IL-11 and IFN-γ. To avoid potential dependency between variables related to multiple lesions from the same person, only a single lesion per patient (selected randomly) was included in the comparative analysis of cytokine expression. Spearman correlation coefficients were calculated (Table 3) and correlation graphs were plotted (Fig. 2). There was a significant positive correlation of cytokine expressions among all cytokines (Table 3).

**Table 3:** Correlation coefficients between cytokine gene expression

Cytokine		IFN-γ	IL-4	IL-11	IL-12p40
IFN-γ	Correlation Coefficient	–	0.564^**^	0.451^**^	0.598^**^
	*p* value	–	< 0.001	0.003	< 0.001
IL-4	Correlation Coefficient	0.564^**^	–	0.830^**^	0.862^**^
	*p* value	< 0.001	–	< 0.001	< 0.001
IL-11	Correlation Coefficient	0.451^**^	0.830^**^	–	0.847^**^
	*p* value	0.003	< 0.001	–	< 0.001
IL-12p40	Correlation Coefficient	0.598^**^	0.862^**^	0.847^**^	–
	*p* value	< 0.001	< 0.001	< 0.001	–

**Correlation is significant at the 0.01 level

### Cytokine gene expression in CL positive patients and in healthy volunteers

**Fig. 3:**
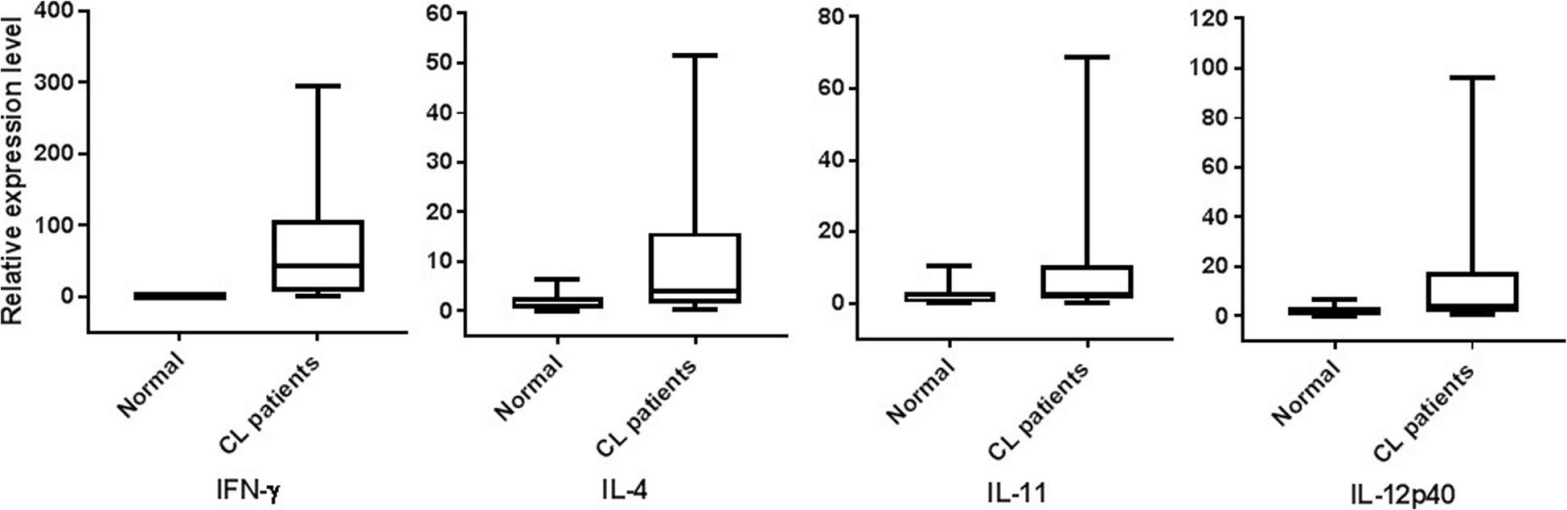
Relative expression levels of cytokine cDNA in CL patients and normal healthy people. Cytokine levels in each group are shown as median and inter quartile range (box) with 5th and 95th percentiles (whiskers)

Cytokine mRNAs were quantified to compare expressions with different clinical presentations. Mann-Whitney U test was used for pair wise comparison of levels of cytokine mRNA between normal healthy people and CL patients. All evaluated cytokine expressions were significantly higher (IFN-γ: *p* <  0.001, IL-4: *p =* 0.002, IL-11: *p =* 0.014 and IL-12p40: *p =* 0.002) in CL lesions compared to healthy volunteers (Fig. 3).

### Correlation between cytokine gene expression and lesion characteristics

**Fig. 4:**
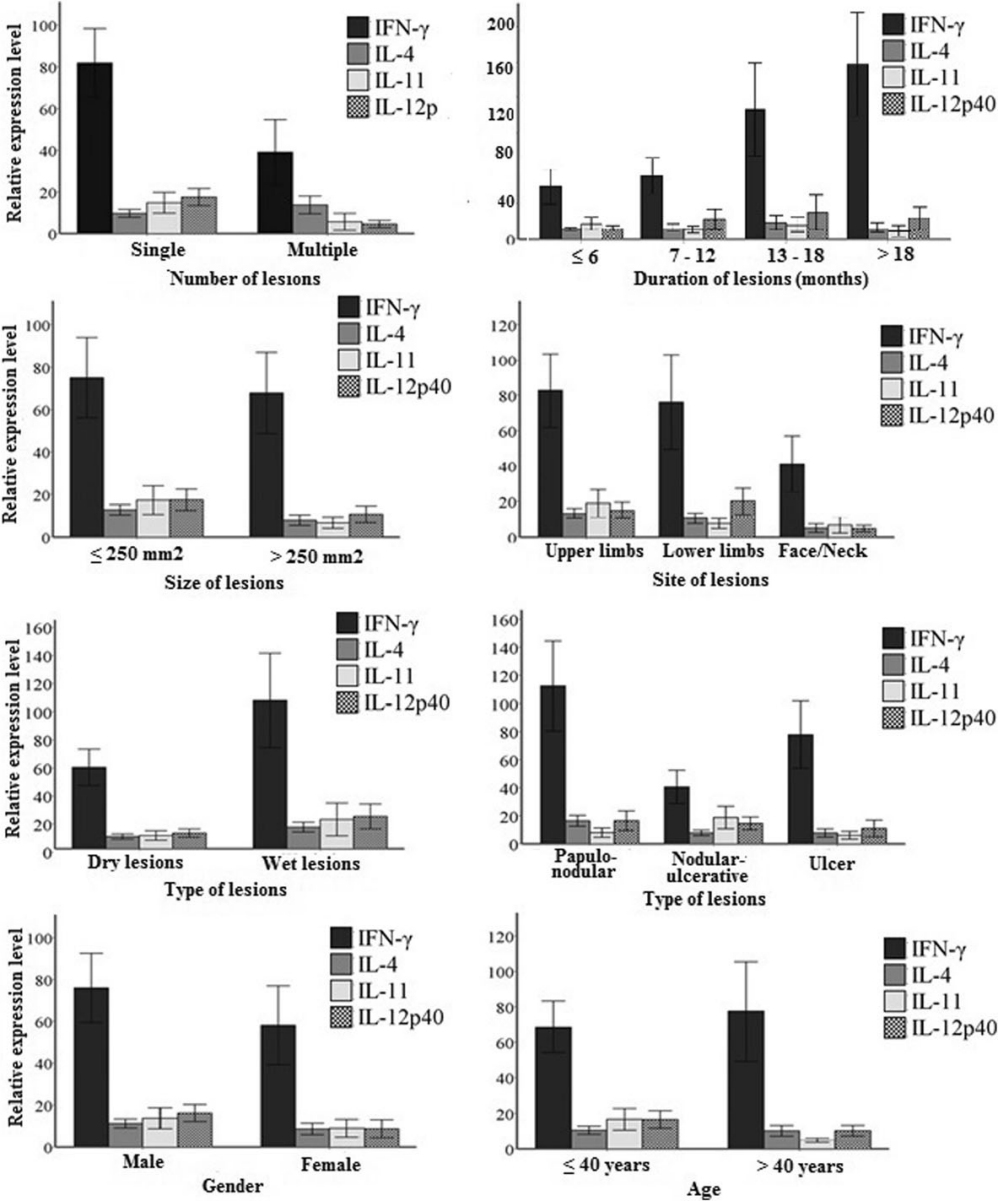
Relative expression levels of cytokine CDNA in CL patients with different clinical characteristics. The results are shown as mean expression values (±) standard errors

Figure 4 shows a relative gene expression with different clinical characteristics. Of the total CL lesions (43), twenty two were less than 6 months duration followed by 9 lesions with 6–12 months, 7 of 12–18 months. Only 5 lesions were more than18 months old. Kruskal – Wallis test was used to determine the association between the expressions of cytokines in relation to time duration. The expression of IFN-γ was the highest compared to the group of cytokines examined. Expression level was significantly higher with longer duration of CL lesions (*p =* 0.021). The expression of IL-4 gradually increased with time and the highest level of expression was observed in 13–18 months old lesions. The peak expression of IL-11 was recorded in 7–12 months old lesions. However, the expressions of IL-4, IL-11 and IL-12p40 were not significantly different in relation to duration of lesions.

Wet CL lesions had significantly high levels of expressions of IL-4, IL-11 and IL-12 p40 (*p* = 0.039, 0.018 and 0.021 respectively). Although the expression of IFN-γ was higher in wet lesions, no significant association was found between IFN-γ and wet/dry type of lesions. The expression of all cytokines is higher in single lesions compared to people having multiple CL lesions. The expressions of IFN-γ were higher in large lesions while the expressions of other cytokines were higher in small lesions. However, no significant association was found between cytokine expressions and number, size and inflammatory signs of lesions. The expressions of all cytokines were high in papulo-nodular lesions compared to other type of lesions. However, IFN-γ expression was significantly higher in papulo-nodular lesions (*p* = 0.023).

### Cytokine gene expression with socio-demographic characteristics

Males showed high levels of IFN-γ and IL-11 expression and females had a higher level of IL-4 expression. The expression of IL-12p40 was comparable in both genders. IFN-γ showed high expression in patients aged over 40 years while the expressions of other cytokines (IL-4, IL-11 and IL-12p40) were higher in patients less than 40 years old. However, those factors were not significantly associated with cytokine expression.

### Modeling and prediction of cytokine expressions and clinical factors

Multiple regression analysis was done to identify predictors associated with the expression of cytokines. Time duration and wet type CL lesions had a significant effect on the expression of IFN-γ. However, there was no significant association between the expressions of other tested cytokines with other clinical parameters (Table 4).

**Table 4:** Multiple regression analysis of cytokine gene expressions and clinical and socio demographic parameters

	IFN- γ		IL-4		IL-11		IL-12p40	
	β (95% CI)	*p* value	β (95% CI)	*p* value	β (95% CI)	*p* value	β (95% CI)	*p* value
Constant	1.518	0.091	0.362	0.111	0.915	0.817	0.937	0.201
R sq	0.338		0.326		0.133		0.286	
Gender	−0.001 (−0.026,0.023)	0.922	− 0.005 (− 0.025, 0.016)	0.649	−0.003 (− 0.029, 0.022)	0.785	−0.006 (− 0.027, 0.014)	0.535
Age	−0.281 (− 0.780,0.219)	0.261	−0.003 (− 0.412, 0.405)	0.988	−0.133 (− 0.645, 0.378)	0.600	−0.065 (− 0.477, 0.348)	0.752
Duration of lesions	0.061 (0.016, 0.105)	0.009	0.017 (−0.020, 0.053)	0.355	0.012 (−0.034, 0.058)	0.595	0.034 (−0.003, 0.071)	0.067
Number of lesions	−0.201 (− 0.712,0.310)	0.420	0.357 (− 0.061, 0.775)	0.092	−0.198 (− 0.721, 0.325)	0.446	−0.166 (− 0.588, 0.256)	0.429
Surface area of lesions	0.001 (0.000,0.003)	0.107	−0.001 (− 0.002, 0.000)	0.143	− 0.001 (− 0.002, 0.001)	0.435	0.000 (− 0.002, 0.001)	0.554
Type of lesions	0.376 (− 0.194, 0.946)	0.189	0.522 (0.056, 0.988)	0.022	0.313 (−0.270, 0.897)	0.282	0.454 (−0.016, 0.925)	0.058
Site of lesions	−0.137 (− 0.472, 0.198)	0.410	− 0.083 (− 0.357, 0.191)	0.543	−0.036 (− 0.379, 0.307)	0.832	−0.006 (− 0.282, 0.271)	0.966
Type of lesions	−0.105 (− 0.430, 0.221)	0.518	−0.176 (− 0.442, 0.090)	0.188	−0.045 (− 0.378, 0.289)	0.787	−0.078 (− 0.347, 0.190)	0.557
Inflammatory signs	−0.129 (− 0.626, 0.367)	0.600	−0.017 (− 0.423, 0.389)	0.934	−0.007 (− 0.515, 0.502)	0.979	−0.165 (− 0.575, 0.245)	0.420

β – Correlation coefficient

## Discussion

The clinical manifestations of leishmaniasis depend on complex interactions between parasite’s invasiveness and host’s immune response [23]. Cytokines are important determinants of host immune system and it may influence the progression or resolution of the disease [24]. In the present study, we quantified the expression of cytokine mRNAs important for macrophage-activation and deactivation in active CL lesions and in healthy skin.

The present study shows the disease progression with various clinical polymorphic forms of CL and its correlation with the host immune response. Furthermore, analysis of cytokine expression showed that the level of different cytokines expression was varied greatly between individual patients and healthy volunteers. Healthy volunteers had very low values of all studied cytokines (IFN-γ, IL-4, IL-11 and IL-12p40), while CL patients had significantly higher values for the same cytokines. This may be due to a high number of macrophages, lymphocytes and epidermal cells present in CL lesions and these cells induce proliferation and activation of effector T cells during CL infection [25]. This emphasizes that the presence of *Leishmania* parasites play a major role in immune response during the pathogenesis of CL caused by *L. donovani*.

In the present study, GAPDH was used as the reference gene. It is one of the most commonly used reference gene for various types of studies. GAPDH is proved to be the most stable among the evaluated genes, and showed no variation between different tissues [26]. This study found a high expression of Th1 response cytokines (IFN-γ and IL-12p40) compared to Th2 type (IL-4) cytokines in CL lesions. This is in agreement with the recent study carried out by Manamperi et al.*,* in 2017 [27]. IL-12 is secreted by antigen-presenting cells (APC). It stimulates the production of IFN-γand regulates Th1 cell differentiation. IFN-γ induces iNOS (inducible nitric oxide synthase) expression and NO production by phagocytes harboring intracellular parasites and it is required for activating macrophages to eliminate parasites and resolve *Leishmania* infection [8, 28]. Furthermore, IFN-γ inhibits the production of cytokines such as IL-4, and IL-10, important cytokines associated with the Type 2 response. Therefore, a high expression of IFN-γ and IL-12 plays an important role in developing a protective immune response against the disease and to prevent dissemination of the disease [29, 30]. Similarly, Ceceres-Dittmar, et al., (1993), have shown the presence of a predominant Th1 response (IFN -γ and IL-12) in LCL patients in Venezuela and weak expression of Th2 cytokines in localized lesions of CL caused by *L. braziliensis* [31].

Expression levels of all cytokines investigated were higher in chronic lesions than in acute CL lesions of *L. donovani* infection. IFN-γ showed the highest expression level in both acute and chronic lesions. In chronic conditions, Th1 cell activates a large number of IFN-γ secreting cells due to a high inflammation and tissue damage which accumulate around the lesion. Therefore, low Th1 response in acute lesions could be a major reason for lesions progression into chronicity. Similarly, Manamperi et al.*,* (2017) have reported increased expression of IFN-γ in non-healing lesions [27]. Generally, IFN-γ is important cytokine in the defense mechanism against the CL. However, over expression of IFN-γ may cause unfavorable response on the host [32].

Studies of the intralesional cytokine expression in acute lesions revealed that IL-4 was expressed at low levels in localized cutaneous lesions [17, 33, 34]. In the present study, slightly higher expression of IL-4 was reported in chronic lesions than in healthy volunteers. However, IL-4 expression was similar in acute and chronic lesions. Previous studies have shown that very low or very high antigen dose could promote a Th2 response, while moderate antigen levels predispose naive cells to become Th1 cells [35]. In the present study, we observed slow growing lesions. This could happen due to high expression of Th1 related cytokines, which cause a reduction in the number of parasites that is reflected in slow growth of the lesions. In addition, early type lesions showed higher expression of IL-4 compared to IL-12. This phenomenon may allow the parasite to survive and multiply in early lesions, leading to progress of the disease. This suggests that high expression level of IFN-γ was not the only cause of long lasting lesions. However, the presence of macrophage inhibitory cytokines such as IL-4 present in the site of infection can cause non-healing chronic lesions. IL-12 also suppresses IL-4 production which is capable of resolution of the infection. High levels of Th1 cell cytokines may affect the low production of IL-4 in these study participants. Louzir, et al., (1998) have found the presence of Th1 and Th2 responses in progressive lesions. A possible explanation provided by authors is the de-activation of macrophages caused by Th2 cytokines thereby causing unfavorable evolution of the lesions [9]. Furthermore, they have also found that the absence of IL-4 contributes to the overall good prognosis of LCL caused by *L. major.* IL-4 was expressed at low levels in localized cutaneous leishmaniasis (LCL) when compared to non-healing lesions of mucocutanoues cutaneous leishmaniasis (MCL) and diffuse cutaneous leishmaniasis (DCL). This finding leads us to speculate that different species of *Leishmania* may have different effects on the host immune system.

Majority of the lesions in this study which gave a prominent Th1 response were single lesions, a lesion size of < 250 mm^2^, papulo-nodular and wet type lesions and duration of < 6 months. According to Awasthi, et al., (2004), a Th1 response is considered to be influenced on a mild or self-curing form of the disease, whereas a Th2 response, which results in the production of IL-4 and IL-10, will influence dissemination of the infection [36]. All CL biopsy samples in this study were from the localized type of lesions and the majority had a Th1 cytokine response possibly limiting dissemination of the disease. In the present study, no significant associations were found between the severity of the disease or the size of ulcers with the expression of Th1 related cytokines. Larger lesions showed a slight elevation of Th2 cytokine (IL4) than IL-12. It may be due to the intensity of host inflammatory reactions in response to parasite burden. This could be the contributing factor behind the high parasite burden found in large lesions. However, no significant difference was found between the level of expression of cytokines and different sizes of the lesions. Previous reports showed that Th2 cytokine are expressed in both healing and non-healing forms of leishmaniasis, however, higher expression levels were reported in chronic and healing stage [33, 34].

Stem cell factor IL-11 is produced by bone marrow stromal cells. IL-11 is shown to stimulate the T-cell-dependent development of immunoglobulin-producing B cells. It acts as a synergistic factor with IL-3, granulocyte-macrophage colony-stimulating factor (GM-CSF) and stem cell factor (SCF) to stimulate proliferation of hematopoietic stem cells and megakaryocyte progenitor cells. High expression of IL-11 in CL lesions emphasizes that a high production of B cells is important to initiate cellular immune against for *Leishmania* parasites.

Dry type lesions were common in this study population, in agreement with previous studies done in Sri Lanka [37]. Abundant parasites and the weak immunity primarily produce dry type lesions while low number of parasites and high immunity make moist type lesions [38]. This was confirmed by the present study as the expression of IL-4 is significantly higher in dry type lesions while IL-12 was significantly higher in moist type lesions. IL-12 is a potent initial stimulus for IFN-γ production and regulates Th1 cell differentiation to kill *Leishmania* parasites. Therefore, IL-12 plays an important role in inducing cell-mediated protection in response to *Leishmania* infection [27]. IL-4 stimulates the differentiation of CD4+ T cells into Th2 cells thereby suppressing Th1 immune responses [39]. The morphological appearances of CL lesions depend on the immunity of the patient and the species of the causative agent. In this study, various types of clinical presentation of CL lesions were found and nodulo-ulcerative lesions were the commonest type, as shown by previous research conducted in Sri Lanka [37]. In the present study, we found a significant low expression of IFN-γ in papulo-nodular lesions. However, a recent study carried out in Sri Lanka was failed to demonstrate this significant relationship [27]. Furthermore, the effect of expression of cytokines on the type of lesions and severity of the disease in different protective responses needs to be investigated.

Cellular immune mechanisms are important factors to control CL [40]. Experimental infection of *Leishmania* spp. in male and female hamsters demonstrated that male animals were more susceptible to infection than female animals [41]. Testosterone has a disease-promoting effect, possibly through a production of Th2 associated anti-inflammatory cytokines such as IL-10 and IL-4 [42] or by blocking a protective effect of estrogen [41]. Furthermore, the present study showed that high expression levels of IL-4 in females and IFN-γ in males. Present study observed high expression of IL-4 in females and IFN-γ in males where as a local study has reported high expression of IL-4 in males [27].

Since biological processes are typically driven by proteins, mRNA expressions are often as a proxy for functional pathway changes, which involve changes in protein levels. This assumes that differences in mRNA levels reflect differences in protein levels [43]. According to Shebl et al., (2010) there was a strong correlation between mRNA expression of IFN-γ and secreted protein levels. In addition, the expression of IL-4 was moderate correlations between the corresponding proteins [44]. Further studies are needed to determine the correlation between the expression of cytokine mRNA and corresponding proteins.

Our results suggest that Th1 response is more prominent compared to the Th2 response in skin lesions of CL patients. However, there are no significant associations between cytokine expression level and number, location and types of lesions.

## Conclusions

Our results suggest that parasite burden may play a vital role in the coordination of an effective immune response during the pathogenesis of CL caused by *L. donovani*. The present study concludes that the expression levels of all cytokines investigated in this study were significantly high in all CL patients and IFN-γ had the highest expression level. Therefore, the results of this study suggest that Th1 response (IFN- γ and IL-12p40) is more prominent than to Th2 response in skin lesions of CL patients in Sri Lanka.

## Abbreviations

APC: Antigen-presenting cells
cDNA: Complementary DNA
CL: Cutaneous leishmaniasis
C_T_: cycle threshold
DCL: Diffuse cutaneous leishmaniasis
DNA: Deoxyribo nucleic acid
GAPDH: glyceraldehyde-3-phosphate dehydrogenase
GM-CSF: Granulocyte-macrophage colony-stimulating factor
IFN-γ: Interferon gamma
IL: Interleukin
iNOS: Inducible nitric oxide synthase
kDNA: kinetoplast DNA
LCL: localized cutaneous leishmaniasis
MCL: Mucocutanoues cutaneous leishmaniasis
mRNA: Messenger ribonucleic acid
ng: Nanograms
NK: natural killer
NO: Nitric oxide
PCR: Polymerase chain reaction
RNA: Ribonucleic acid
RT-PCR: Real time polymerase chain reaction
SCF: Stem cell factor
SD: Standard deviation
SE: Standard error
Th: T helper
μg: microgram
μl: microliter
